# Osteopathic care for spinal complaints: A systematic literature review

**DOI:** 10.1371/journal.pone.0206284

**Published:** 2018-11-02

**Authors:** Nick Verhaeghe, Janne Schepers, Patrick van Dun, Lieven Annemans

**Affiliations:** 1 Department of Public Health, Interuniversity Centre for Health Economics Research (I-CHER), Ghent University, Ghent, Belgium; 2 Department of Public Health, Interuniversity Centre for Health Economics Research (I-CHER), Vrije Universiteit Brussel, Brussels, Belgium; 3 Department of Pharmaceutical and Pharmacological Sciences, KU Leuven, Leuven, Belgium; 4 Commission for Osteopathic Research, Practice and Promotion vzw (CORPP), National Centre of COME Collaboration, Mechelen, Belgium; Stanford University School of Medicine, UNITED STATES

## Abstract

The aim of the current study was to evaluate the literature examining the impact of osteopathic care for spinal complaints. The bibliographic databases Medline (Pubmed), Web of Science, Embase, and PEDro were searched. In addition, a number of grey literature sources were searched. Only randomized controlled trials conducted in high-income Western countries were considered. Two authors independently screened the titles and abstracts. Primary outcomes included ‘pain’ and ‘functional status’, while secondary outcomes included ‘medication use’ and ‘health status’. It was examined if differences existed related to the treatment protocol and geography (European vs. US studies). Study quality was assessed using the risk of bias tool of the Cochrane Back Review Group. Nineteen studies were included and qualitatively synthesized. Nine studies were from the US, followed by Germany with seven studies. The majority of studies (n = 13) focused on low back pain. In general, mixed findings related to the impact of osteopathic care on primary and secondary outcomes were observed. For the primary outcomes, a clear distinction between US and European studies was found, in favor of the latter ones. Studies were characterized by substantial methodological differences in sample sizes, number of treatments, control groups, and follow-up. In conclusion, there is some evidence suggesting that osteopathic care may be effective for people suffering from spinal complaints. Further studies with larger study samples and assessment of long-term impact are required to further increase the evidence-based knowledge of the potential of osteopathic care for individuals suffering from spinal complaints.

## Introduction

Spinal complaints including low back pain (LBP) and neck pain are common conditions. The epidemiology of these conditions is well studied. For LBP, lifetime prevalence of non-specific LBP is estimated to be about 84%, with the prevalence of chronic LBP about 23% [1]. Neck pain annual prevalence rates are estimated to range between 15% and 50% [2]. Spinal complaints are thus a frequent health problem and they are associated with considerable medical, social and economic consequences such as the need for medical care, limitations in activities, absence from work, and disability [3–6]. It is estimated that in less than 15% of LBP cases, a specific cause can be identified [7]. As a consequence, the origin of LBP and neck pain is often unknown meaning that it is not possible to identify a specific cause of the pain and as a consequence the complaints are considered ‘non-specific’ [8]. Guidelines on the treatment of spinal complaints include a variety of approaches such as exercise, cognitive behavioral therapy, multidisciplinary treatment, and spinal manipulation [9–11].

Manual therapy/medicine including osteopathy is a common treatment option for musculoskeletal complaints including LBP and neck pain, and its use has worldwide increased in the past decades [12]. Osteopathy was founded at the end of the 19^th^ century in the United States (US) and introduced in Europe at the beginning of the 20^th^ century. International differences in osteopathic healthcare regulation influence its scope of practice, which may explain some of the differences between US and European and Australasian osteopathic healthcare provision [12, 13]. Osteopathy can be defined as a primary contact and patient-centered healthcare discipline, that emphasizes the interrelationship of structure and function of the body, facilitates the body’s innate ability to heal itself, and supports a whole-person approach to all aspects of health and healthy development, principally by the practice of manual treatment [14]. The findings of previous literature reviews [15–18] assessing the impact of osteopathic care for spinal complaints suggested clinically relevant effects for LBP and neck pain on reducing pain and improving functional status. In all reviews, it was concluded that the findings of the studies must be cautiously interpreted due to methodological considerations such as small sample sizes, lack of long-term follow-up, and differences in comparison groups. The aim of the current literature review was to evaluate published randomized controlled trials examining the effectiveness of care administered by osteopathic practitioners for improving pain and functional status in patients with spinal complaints as compared to control treatments. Conversely to the previous reviews focusing on a specific spinal area such as LBP [15, 17, 18] or neck pain [16], the current review focused on spinal complaints including back pain, LBP, neck pain, and pelvic girdle pain. In addition, conversely to the reviews by Franke et al. [16] and Orrock and Myers [18], no restrictions were made concerning the chronicity of the complaint. Finally, conversely to previous reviews, an analysis distinguishing between different osteopathic treatment protocols is considered.

## Materials and methods

**Fig 1:**
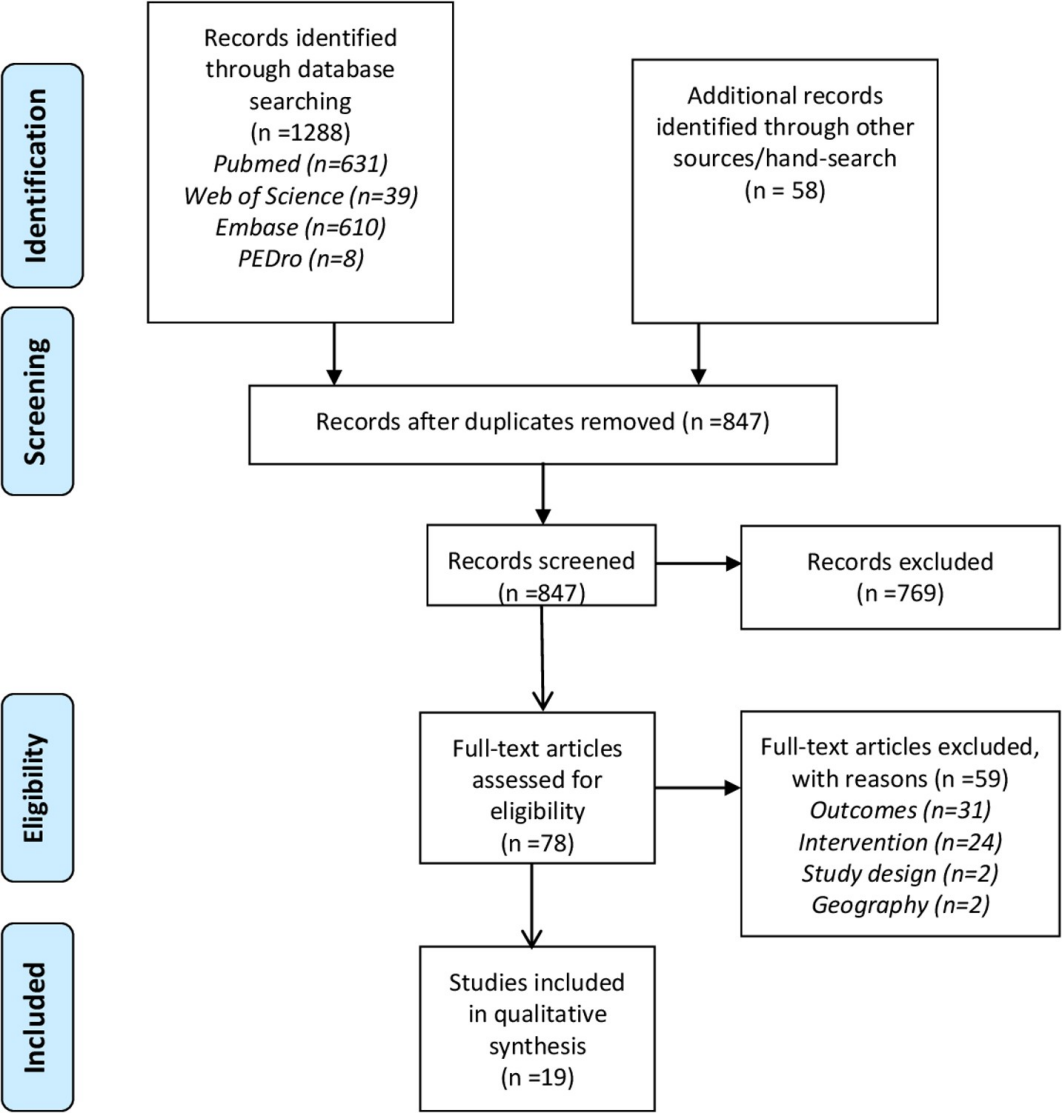
Flow chart of the study selection process.

The study followed the ‘Preferred Reporting Items for Systematic Reviews and Meta-Analyses (PRISMA)’ (S1 Table) [19]. A comprehensive literature review was conducted searching the peer-reviewed bibliographic databases Medline (Pubmed), Web of Science (Core Collection), Embase, and PEDro. For each database, a search algorithm was developed adapted to the specific requirements or features of the database using a combination of the terms ‘low back pain’ [MeSH], ‘back pain’ [MeSH], ‘neck pain’ [MeSH], ‘pelvic pain’ [MeSH], ‘pelvic girdle pain’ [MeSH], ‘spine’ [MeSH], ‘manipulation, osteopathic’ [MeSH], ‘manipulation, spinal’ [MeSH], ‘osteopathic medicine’ [MeSH], ‘comparative effectiveness research’ [MeSH], ‘pragmatic clinical trial’ [MeSH], ‘osteopathy’, ‘osteopathic’, ‘manipulative’, ‘manipulation’, ‘spinal’, ‘effectiveness’, and ‘efficacy’. The databases were searched referring to the years 1995 (01/01) until 2018 (31/05). In addition, previous published literature reviews [15–18, 20] were searched to identify studies missed by our search. In the review by Franke et al. [15], a number of studies derived from the grey literature source www.osteopathic-research.com were also included. To be as comprehensive as possible, we also searched this website to identify additional records. In addition, the grey literature sources ‘OAIster’ (https://www.oclc.org/en/oaister.html) and ‘OpenGrey’ (http://www.opengrey.eu/) were searched using terms such as ‘low back’, ‘neck’, ‘spine’, ‘osteopathy’, ‘osteopathic’, ‘trial’, ‘effectiveness’. The initial search yielded 1,346 records. After excluding the duplicates (n = 499), 847 records remained for further evaluation (Fig 1).

Eligibility criteria were defined using the PICOS (Population, Intervention, Comparator, Outcome, and Study design) strategy [21]. An overview of the eligibility criteria is listed in Table 1.

**Table 1:** Eligibility criteria.

PICOS	inclusion criteria	exclusion criteria
**population**	individuals with back pain, LBP, neck pain, pelvic girdle pain–no age restrictions	individuals suffering from other than spinal complaints
**intervention**	care administered by an osteopathic practitioner	care performed by other professionals
**comparator**	no intervention, sham/placebo, or usual care (no restrictions on definition of usual care)	NA
**outcomes**	pain, functional status, medication use, health status	other outcomes
**study design**	randomized controlled trials	other study designs
**language**	English, French, Dutch, German	other languages
**time period**	published from 1/1/1995–31/01/2017 (Pubmed & Web of Science)	published prior to 1/1/1995 and after 31/01/2017
**geography**	high-income Western countries	other

LBP, low back pain; NA, not applicable

The titles and/or abstracts were independently screened on the inclusion and exclusion criteria by two authors (NV and JS). Variations in authors’ opinion were resolved through discussion and consensus involving the other authors (LA and PvD).

### Data analysis

The effect of osteopathic care on the primary outcomes ‘pain’ and ‘functional status’ was assessed considering four levels of effectiveness including (from poorest to best): (1) the outcome is found to be numerically or significantly worse from baseline to endpoint within the osteopathic care group or is found to be worse than in the comparison group, (2) there is no significant between-group difference, but the outcome is found to be numerically improved in the osteopathic care group compared to baseline, (3) there is no significant between-group difference, but the outcome is found to be significantly improved in the osteopathic care group compared to baseline, and (4) there is a significant difference between the osteopathic care group and the comparison group. Inspiration for choosing this classification was derived from a systematic review and meta-analysis of quality of care in for-profit and not-for-profit nursing homes [22]. It was examined if differences in level of effectiveness were found according to the treatment protocol. A distinction was made between ‘custom tailored’ (the determination of the treatment was left at the clinical judgement of the osteopathic practitioner), ‘semi-standardized’ (where the osteopathic practitioner could choose from a set of predefined manual techniques, or it included a single or a set of standardized manual technique(s) always to be offered along with free choice of manual techniques left at the clinical judgment of the practitioner), and ‘standardized’ (where the treatment protocol included a detailed description of manual techniques to be applied) treatment protocols. It was also examined if differences in impact of osteopathic care on pain and functional status existed between studies conducted in Europe vs. those conducted in the US. The secondary outcomes included the effect of osteopathic care on ‘medication use’ since this is an often used treatment option [23] and on ‘health status’ since spinal problems have a considerable impact on health-related quality of life [24, 25].

### Assessment of risk of bias

The assessment of risk of bias was undertaken by one researcher (NV). In case of any doubt, this was resolved through discussion and consensus involving another author (PvD). The risk of bias in individual studies was assessed using a checklist of 12 criteria as recommended by the Cochrane Back and Neck Group [26]. For each study, the risk of bias criteria were scored ‘yes’ (criterion met), ‘no’ (criterion not met) or ‘unsure’. The latter score was used in case information on the criterion was missing or not clear. A study was rated as having a ‘low risk of bias’ when at least six criteria were met. Contrary, a study was rated as having a ‘high risk of bias’ when fewer than six of the 12 criteria were met.

## Results

### Study selection process

Seven hundred and sixty-nine records were excluded on title and abstract screening. So, 78 records remained of which the full-text was evaluated. Fifty-nine records (reasons: outcome, n = 31; intervention, n = 24; study design, n = 2; geography, n = 2) were excluded after the full-text evaluation resulting in 19 records included (Fig 1).

### Study characteristics

Nine studies were conducted in the US [27–35], and seven in Germany [36–42]. Studies originating from the UK [43, 44] and Italy [45] made up the remaining studies (Table 2). In the majority of studies (n = 13), osteopathic care was aimed at people suffering from LBP (Table 2). The studies differed considerably related to the study populations based on age-groups, duration of the spinal complaints, or target groups such as ‘obese females’ [45], ‘women post-partum’ [36, 40] or ‘diabetes mellitus patients’ [32]. The number of treatment sessions varied from a single session [35] to ten sessions [45] (Table 2). In 13 studies [28, 31–34, 36–38, 40–44], a follow-up period was considered ranging from two weeks [42] to nine months [43, 44] (Table 2). The number of study participants receiving osteopathic care ranged from ten [45] to 353 [44] (Table 3). The osteopathic treatment protocol could be labelled as ‘standardized’ in one study [30], ‘semi-standardized’ in nine studies [28, 29, 31–33, 35, 43–45], and ‘custom tailored’ in nine studies [27, 34, 36–42] (Table 2). A wide variety of comparators across studies was observed (Table 3).

**Table 2:** Overview of characteristics of included studies.

first author (year)	country	study population	osteopathic intervention	intervention duration	follow-up*
Andersson (1999)	US	patients 20-59y LBP ≥3w-≤6m	custom tailored	12w (8 sessions)	NA
Licciardone (2003)	US	patients 21-69y non-specific LBP ≥3m	custom tailored	5m (7 sessions)	4w
UK BEAM (2004)	UK	patients LBP	semi-standardized	12w (8 sessions)	9m
McReynolds (2005)	US	patients neck pain <3w	semi-standardized	one session	NA
Peters (2006)	Germany	pregnant (20-30w) women LBP	custom tailored	4w (4 sessions)	NA
Heinze (2006)	Germany	patients 18-65y LBP ≥3w-≤6m	custom tailored	6w (2–3 sessions)	6w
Chown (2008)	UK	patients 18-65y >3m simple LBP	semi-standardized	3m (5 sessions)	9m
Recknagel (2008)	Germany	women 18-46y post-partum BP ≥3m-≤24m	custom tailored	6w (4 sessions)	6w
Schwerla (2008)	Germany	patients 20-55y ≥3m non-specific neck pain	custom tailored	10w (5 sessions)	3m
Engemann (2009)	Germany	patients 18-60y chronic non-specific neck pain	custom tailored	6 sessions**	3m
Licciardone (2010)	US	pregnant women (≤30w) BP	semi-standardized	10w (7 sessions)	NA
Cruser (2012)	US	military staff 18-35y acute LBP	semi-standardized	4w (4 sessions)	4w
Vismara (2012)	Italy	obese females (BMI>30kg/m^2^chronic LBP >6m	semi-standardized	10 sessions**	NA
Licciardone (2013a)	US	diabetes mellitus patients 21-69y LBP	semi-standardized	8w (6 sessions)	4w
Licciardone (2013b)	US	non-pregnant adults 21-69y LBP ≥3m	semi-standardized	8w (6 sessions)	4w
Belz (2015)	Germany	women 18-42y post-partum non-specific BP ≥3m	custom tailored	10w (5 sessions)	3m
Hensel (2015)	US	pregnant (30w) women 18-35y LBP	standardized	10w (7 sessions)	NA
Schwerla (2015)	Germany	women 18-42y post-partum LBP or pelvic girdle pain ≥3m	custom tailored	6w (4 sessions)	2w
Licciardone (2016)	US	patients 21-69y non-specific LBP ≥3m	semi-standardized	8w (6 sessions)	4w

*time period starting after the last treatment session

**no information on intervention duration provided

BP, back pain; LBP, low back pain; m, months; NA, not applicable; w, weeks; y, year

**Table 3:** Overview of intervention and control groups, outcome measurements and main findings.

first author (year)	intervention + n° of participants	control + n° of participants	outcome measurement	findings intervention vs. control
Andersson (1999)	OT + SAT (n = 83)	SAT (n = 72)	pain: VAS; functional status: RMDQ/OPQ	**pain**: 32.0±23.0 mm vs. 26.3±24.1 mm (p = 0.19); **functional status**: median: 5 vs. 5 (p = 0.16); **medication**: anti-inflam.:24.3% vs. 54.3%(p<0.001), muscle relaxants:6.3% vs. 25.1%(p<0.001)
Licciardone (2003)	OT (n = 48)	sham manipulation (n = 23) no intervention (n = 20)	pain: VAS, functional status: RMDQ	**pain**: OT vs. control at 1m (p = 0.01), 3m (p = 0.001), 6m (p = 0.02) / OT vs. sham at 1m (p = 0.29), 3m (p = 0.96), 6m (p = 0.95); **functional status**: no significant differences among treatment groups; **medication**: no significant between-group differences
UK Beam (2004)	OT+best care* (n = 353) OT+best care*+exercise (n = 333)	best care* (n = 338)	functional status: RMDQ	OT+best care vs. best care: 3m, 1.57 [95%CI 0.82–2.32] p = 0.001; 12m, 1.01 [95%CI 0.22–1.81] p = 0.05 / OT+best care+ exercise vs. best care: 3m, 1.87 [95%CI 1.15–2.60] p = 0.001; 12m, 1.30 [95%CI 0.54–2.07] p = 0.01
McReynolds (2005)	OT (n = 29)	medication (n = 29)	pain: NRS/PRS	2.8±1.7 vs. 1.7±1.6 (p = 0.02); **pain relief**: no significant between-group change (p = 0.10)
Peters (2006)	OT (n = 30)	no intervention (n = 30)	pain: VAS; functional status: QBPDS	**pain**: 4.4±2.4vs.-0.3±1.9(p<0.0005); improvement: OT,67%; control, 4.5% deterioration; **functional status**: 11.1±16.6 vs. -8.4±10.4 (p<0.0005) improvement: OT, 28%; control, 20% deterioration
Heinze (2006)	OT + heat + physiotherapy (n = 28)	heat + physiotherapy (n = 32)	pain: NRS; functional status: RMDQ	**pain**: 4.3 vs. 1.8 (p<0.001) improvement: OT, 66%; control: 30%; **functional status**: 8.9 vs. 3.4 (p<0.001) improvement: OT, 73%; control, 30%
Chown (2008)	OT (n = 79)	physiotherapy (n = 80)	functional status: ODI	mean score OT, -5.0 [95%CI -1.6, -8.4] p<0.01; PT, -4.1 [95%CI -1.4, -6.9] p<0.01
Schwerla (2008)	OT + sham UST (n = 23)	UST (n = 18)	pain: NRS	average pain, 2.48 vs. 0.75 difference: -1.73 [95%CI -3.13, -0.32] p = 0.017 / OT: significant decrease in actual (p = 0.001) and worst (p = 0.001) pain
Recknagel (2008)	OT (n = 20)	no intervention (n = 19)	pain: VAS; functional status: OPQ	**pain**: diff. 4.6 [95%CI 3.4,-5.8] p<0.001 improv.: OT, 4.8 [95%CI 3.7,5.9)p<0.001 70% improvement; **funct. status**: diff. 1.8[95%CI 1.2,2.4] p<0.001 improv.: OT, 1.8 [95%CI 1.2,2.3] p<0.001
Engemann (2009)	OT (n = 15)	no intervention (n = 15)	pain: NPAD/NRS	-2.14 vs. 0.32 (p<0.001)
Licciardone (2010)	OT + UOC (n = 49)	UOC + sham UST (n = 48) / UOC (n = 49)	pain: VAS; functional status: RMDQ	**pain**: no significant between-group differences or significant improvement in OT; **functional status**: OT, deterioration from baseline to treatment end
Cruser (2012)	OT + UC (n = 30)	UC (n = 30)	pain: VAS; functional status: RMDQ	**pain**: pain now, 1.96±1.47 vs. 3.73±2.39 difference: -1.47 [95%CI -2.75, -0.18] p = 0.026; **functional status**: no significant between-group difference / OT (baseline-study end): 12.37±5.30 vs. 4.44±5.95 (p<0.001); **medication**: no significant between-group differences
Vismara (2012)	OT + exercise (n = 10)	exercise (n = 11)	pain: VAS; functional status: RMDQ/ODI	**pain**: 1.4±1.2 vs. 3.0±0.8 (p<0.05); **functional status**: RMDQ: 3.13±2.85 vs. 7.27±2.19 (p<0.05) / ODI: 3.50±1.69 vs. 9.18±3.34 (p<0.05)
Licciardone (2013a)	OT + (sham) UST (n = 19)	shamOT+(sham)UST(n = 15)	pain: VAS	difference 1.7 [95%CI 3.2, 0.1] p = 0.04
Licciardone (2013b)	OT + (sham) UST (n = 230)	sham OT + (sham) UST (n = 225)	pain: VAS; functional status: RMDQ	**pain**: -1.8(IQR-3.1,0.0)vs.-0.9(IQR-2.5,0.3) p = 0.002/moderate improv.: 63% vs. 46% RR = 1.38 [95%CI 1.16, 1.64] p<0.001 / substantial improv.: 50% vs. 35% RR = 1.41 [95%CI 1.13, 1.76] p = 0.002 ; **funct. status **: no signif.diff./significant improv.in OT; **medication**: 13% vs. 20% (p = 0.048)
Hensel (2015)	OT (n = 136)	placebo UST (n = 131) / UOC (n = 133)	pain: VAS; functional status: RMDQ	**pain**:mean OTvs.UST0.15[95%CI-3.07,3.36]p>0.999; OTvs.UOC-7.11[95%CI-10.30,-3.93]p<0.001; **funct. status**: OTvs.UST 0.21[95%CI-.73,1.14] p>0.999; OTvs.UOC: -2.25[95%CI -.18,-1.32]p<0.001
Belz (2015)	OT (n = 30)	no intervention (n = 30)	pain: VAS; functional status: PGQ	**pain**: -4.2±2.0 vs. -0.4±1.3 difference 3.8 [95%CI 2.8, 4.7) p<0.0005; **functional status**: -22.7±10.1 vs. -8.8±15.4 difference 13.9 [95%CI 6.7, 21.0] p<0.0005
Schwerla (2015)	OT (n = 40)	no intervention (n = 40)	pain: VAS; functional status: ODI	**pain**: -5.3±1.7 vs. -0.5±1.2 difference 4.8 [95%CI 4.1, 5.4] p<0.001; **functional status**: -12.6±6.5 vs. -2.0±5.2 difference 10.6 [95%CI 8.0, 13.2] p<0.001
Licciardone (2016)	OT (n = 175)	sham OT (n = 170)	pain: VAS; functional status: RMDQ	**pain**: median (IQR) reduction 2.0 (0.2, 3.6) vs. 1.2 (-0.5, 2.5) p = 0.002; **functional status**: median (IQR) reduction 2 (0, 5) vs. 2 (0, 4) p = 0.66

*based on the UK national acute back pain guidelines 'continuing normal activities and avoiding rest' / IQR, interquartile range; NPAD, Neck Pain and Disability Scale; NRS, Numerical Rating Scale

ODI, Oswestry Disability Index; OPQ, Oswestry Pain Questionnaire; OT, osteopathic treatment; PGQ, Pelvic Girdle Pain Questionnaire; PRS, Pain Relief Scale; QBPDS, Quebec Back Pain Disability Scale; RMDQ, Roland-Morris Disability Questionnaire; RR, response ratio; SAT, standard allopathic treatment; UC, usual care; UOC, usual obstetrical care; UST, ultrasound treatment; VAS, Visual Analogue Scale

### Primary outcome–pain

The impact of osteopathic care on pain was assessed in 17 studies (Table 4) applying different measurement instruments including the VAS [27, 28, 30–34, 39, 40, 45], the NRS [35, 37, 38, 41], the PRS [35], and the NPAD [37]. From a treatment protocol point of view, a ‘custom tailored’ protocol was applied in nine studies. Seven showed significant between-group differences in favor of the osteopathic care group compared to controls: Peters & Van der Linde [39] (between-group difference VAS: 4.7, p<0.0005), Heinze [38] (NRS: 2.5, p<0.001), Recknagel & Roß [40] (VAS: 4.6, p<0.001), Schwerla et al. [2008] [41] (NRS: average pain 1.7, p = 0.017), Schwerla et al. [2015] [42] (VAS: 4.8, p<0.001), Engemann & Hofmeier [37] (NPAD/NRS: 2.5, p<0.001), Belz et al. [36] (VAS: 3.8, p<0.0005) and Licciardone et al. [2003] [34] (osteopathic treatment vs. no intervention VAS: no exact figures reported). In those applying a ‘(semi-)standardized’ protocol, osteopathic care was found to be effective compared to controls in five of eight studies (Table 4): Cruser et al. [28] (between-group difference: VAS: 1.5, p = 0.026), Vismara et al. [45] (VAS: 1.6, p<0.05), Licciardone et al. [2013a] [32] (1.7, p = 0.04), Licciardone et al. [2013b] [33] (0.9, p = 0.002), and Licciardone et al. [2016] [31] (0.8, p = 0.002). In all European studies (n = 8), significant differences in pain improvement in individuals receiving osteopathic care compared to controls were observed. In seven of these studies, a ‘custom tailored’ protocol was used (Table 4). In the US studies applying such a protocol (n = 2), the findings were less homogeneous. In Andersson et al. [27], an only non-significant pain improvement, measured using the VAS, of 3.2±2.3 points (p = 0.19) from baseline to treatment end was observed in the osteopathic care group. In Licciardone et al. [34], back pain significantly improved in those receiving osteopathic care compared to those receiving no intervention (p = 0.02), but not compared to participants receiving sham manipulation (p = 0.95). Overall, the most promising results in the US studies, i.e. merely significant differences between the osteopathic care group and the comparison groups were identified in only four of nine studies including Cruser et al. [28] (between-group difference: VAS: 1.5, p = 0.026), Licciardone et al. [2013a] [32] (1.7, p = 0.04), Licciardone et al. [2013b] [33] (0.9, p = 0.002), and Licciardone et al. [2016] [31] (0.8, p = 0.002) (Table 4).

**Table 4:** Effectiveness of osteopathic care related to the outcome ‘pain’.

Study	Treatment protocol	treatment effect			
		OT worse than controls	non-significant improvement OT	signif. improvement OT/no signif. between-group difference	significant between-group difference
**EUROPEAN STUDIES**					
Peters (2006)	custom				x
Heinze (2006)	custom				x
Recknagel (2008)	custom				x
Schwerla (2008)	custom			actual & worst pain	average pain
Engemann (2009)	custom				x
Schwerla (2015)	custom				x
Belz (2015)	custom				x
Vismara (2012)	semi-standardized				x
**US STUDIES**					
Andersson (1999)	custom		x		
Licciardone (2003)	custom		OT vs. sham		OT vs. control
McReynolds (2005)	semi-standardized		pain relief		pain intensity
Cruser (2012)	semi-standardized				x
Licciardone (2013a)	semi-standardized				x
Licciardone (2013b)	semi-standardized				x
Hensel (2015)	semi-standardized			OT vs. PUT	OT vs. UOC
Licciardone (2016)	semi-standardized				x
Licciardone (2010)	standardized		x		

OT, osteopathic treatment; PUT, placebo ultrasound treatment; UOC, usual obstetrical care

### Primary outcome–functional status

The impact of osteopathic care on functional status was examined in 15 studies (Table 5) applying different measurement tools including the RMDQ [27–31, 34, 38, 44–46], the ODI [42, 43, 45], the OPQ [27, 40], the QBPDS [39], and the PGQ [36]. In none of three studies focusing on the neck area [35, 37, 41], the impact of osteopathic care on functional status was assessed. In seven of eight European studies, osteopathic care was found to significantly improve functional status compared to controls: Peters & Van der Linde [39] (between-group difference QBPDS: 19.5, p<0.0005), Heinze [38] (RMDQ: 5.5, p<0.001), Recknagel & Roß [40] (OPQ: 1.8, p<0.001), Schwerla et al. [42] (ODI: 10.6, p<0.001), Belz et al. [36](PGQ: 13.9, p<0.0005), UK BEAM Trial Team [44] (RMDQ: OT + best care vs. best care: 1.01, p = 0.05; OT + best care + exercise vs. best care: 1.30, p = 0.001), and Vismara et al. [45] RMDQ: 4.14, p<0.05; ODI: 5.68, p<0.05). In the study by Chown et al. [43], improvements in functional status were observed in individuals receiving osteopathic care (mean -5.0, 95%CI: -1.6, -8.4, p<0.01), as well as in those receiving physiotherapy (mean -4.1, 95%CI: -1.4, -6.9, p<0.01). In all five European studies applying a ‘custom tailored’ treatment protocol, significant between-group differences in favor of osteopathic care were found, while this was not the case in the US studies applying such a protocol (Table 5). Overall in the US studies, osteopathic care was found to significantly improve functional status in only one study [29]. In that study, functional status significantly improved in patients receiving osteopathic care compared to those receiving usual obstetrical care (between-group difference measured by the RMDQ 2.25, p<0.001), but not compared to patients receiving placebo ultrasound therapy (0.21, p>0.999) (Table 5). In one US study [30], functional status at the end of treatment even deteriorated in the osteopathic care group (Table 5).

**Table 5:** Effectiveness of osteopathic care related to the outcome ‘functional status’.

Study	Treatment protocol	treatment effect			
		OT worse than controls	non-significant improvement OT	signif. improvement OT/no signif. between-group difference	significant between-group difference
**EUROPEAN STUDIES**					
Peters (2006)	custom				x
Heinze (2006)	custom				x
Recknagel (2008)	custom				x
Schwerla (2015)	custom				x
Belz (2015)	custom				x
UK BEAM (2004)	semi-standardized				x
Chown (2008)	semi-standardized			x	
Vismara (2012)	semi-standardized				x
**US STUDIES**					
Andersson (1999)	custom		x		
Licciardone (2003)	custom		x		
Cruser (2012)	semi-standardized			x	
Licciardone (2013b)	semi-standardized		x		
Hensel (2015)	semi-standardized			OT vs. PUT	OT vs. UOC
Licciardone (2016)	semi-standardized		x		
Licciardone (2010)	standardized	x			

OT, osteopathic treatment; PUT, placebo ultrasound treatment; UOC, usual obstetrical care

### Secondary outcome–medication use

Information on the impact of osteopathic care on medication use was examined in four studies [27, 28, 33, 34] (Table 3). In the study by Cruser et al. [28], information on medication use was obtained from records, while in the study by Licciardone et al. [2013b] [33], this information was collected from patients or from community-based clinicians. In the two other studies [27, 34], no detailed information how data on medication use was collected was provided. In Andersson et al. [27], significant differences in prescription practices between the osteopathic care group and a control group receiving standard allopathic care were observed for anti-inflammatory drugs (24.3% vs. 54.3%, p<0.001), and muscle relaxants (6.3% vs. 25.1%, p<0.001). Licciardone et al. [34] identified no significant differences in back-specific medication use between the osteopathic care group and a control group receiving sham manipulation. In another study by Licciardone et al. [33], a significant difference in the use of prescription drugs for LBP was found between the study population receiving osteopathic care and those receiving sham manipulation (13.0% vs. 20.0%, p = 0.048). Finally, Cruser et al. [28] identified no significant differences in medication prescribing practices between patients in osteopathic care and those receiving usual care for LBP.

### Secondary outcome–health status

The impact of osteopathic care on health status was evaluated in six studies [33, 34, 37, 41, 43, 44]. Licciardone et al. [2003] [34] found significantly better scores for physical functioning and mental health (measured by the SF-36) one month after treatment in the osteopathic care group compared to the no intervention group. Compared to sham manipulation, significant between-group differences were only identified for physical functioning, but not for mental health. In another study by Licciardone et al. [2013b] [33], no significant effects on health status (measured by the SF-36) were observed (mean [IQR] OT vs. sham OT: 72 [52–87] vs. 72 [57–87], p = 0.87). In the UK BEAM trial [44] the effect of osteopathic care in addition to ‘best care’ on health-related quality of life was measured by the SF-36. A significant between-group difference (osteopathic care + best care vs. best care) was found for the physical component score at the end of a three-month treatment period (between group difference: 2.52 [95%CI: 1.30, 3.74], p<0.001) and after nine months of follow-up (1.68 [95%CI: 0.18, 3.19], p<0.05). For mental health, this was only observed at the end of the treatment phase (2.88 [95%CI: 1.36, 4.40], p<0.001). In Chown et al. [43], health-related quality of life (measured by the EQ-5D) significantly improved in individuals receiving osteopathic care (mean difference at 6 weeks: 0.11 [95%CI: 0.02, 0.19], p<0.05) as well as in the physiotherapy group (mean difference at 6 weeks: 0.10 [95%CI: 0.01, 0.18], p<0.05). In Schwerla et al. [41], a significant difference in bodily pain (measured by the SF-36) was identified in the study population receiving osteopathic care and in those receiving sham ultrasound (between-group difference: 14.6 [95%CI: 2.58, 26.60], p = 0.019). Finally, in the study by Engemann & Hofmeier [37], a significant-between group difference was observed between the osteopathic care group and the no intervention group for the physical component (between-group difference: 8.7, p = 0.012) and mental component score (7.5, p = 0.05).

### Risk of bias

All 19 studies had a low risk of bias, meaning that at least six of the criteria were met (Table 6). In none of the studies blinding of patients, care provider or outcome assessor occurred or it was unclear. Important to note is that in three studies [29, 38, 42] the study groups differed to some extent at baseline. In Schwerla et al. [42], pain intensity at baseline (measured by the Oswestry Disability Index) was significantly different between the osteopathic care group and the controls (16.8±6.7 vs. 22.1±7.2; p = 0.001). In the study by Heinze [38], a significant between-group difference in Numerical Rating Scale score was observed (intervention vs. control: 6.5 vs. 5.9; p = 0.04). In Hensel et al. [29], subscale pain scores (measured by the Quadruple Visual Analog Scale) for ‘pain now’ and ‘pain best’ significantly differed between the three study groups (osteopathic care, ultrasound placebo, and usual care). In the latter study, a linear mixed model suitable for repeated measures was applied and models were adjusted for baseline values to address differences in baseline pain and disability. In the studies by Schwerla et al. [42] and Heinze [38] no information if the authors did control for the differences at baseline was reported.

**Table 6:** Risk of bias in the individual studies included in the review.

first author (year)	randomi-zation?	allocation concealed?	patient blinding?	care provider blinding?	outcome assessor blinding?	drop-outs described & accepatble?	ITT-analysis?	free of suggestion of selective outcome reporting?	groups similar at baseline?*	co-interventions avoided or similar?	acceptable compliance?	similar timing outcome assessment?	yes-score
Andersson (1999)	yes	yes	no	no	no	no	no	yes	yes	yes	yes	yes	7
Licciardone (2003)	yes	yes	U	no	U	yes	U	no	yes	yes	U	yes	6
UK BEAM (2004)	yes	yes	no	no	no	yes	no	yes	yes	yes	yes	yes	8
McReynolds (2005)	yes	yes	no	no	no	yes	U	yes	yes	yes	NA**	yes	7
Peters (2006)	yes	yes	no	no	no	yes	no	yes	yes	yes	yes	yes	8
Heinze (2006)	yes	yes	no	no	no	yes	yes	yes	no	yes	yes	yes	8
Chown (2008)	yes	yes	U	no	U	yes	U	yes	yes	yes	yes	yes	8
Recknagel (2008)	yes	yes	no	no	no	yes	yes	yes	yes	yes	yes	yes	9
Schwerla (2008)	yes	yes	no	no	no	yes	no	yes	yes	yes	U	yes	7
Engemann (2009)	yes	yes	no	no	no	yes	yes	yes	yes	yes	U	yes	8
Licciardone (2010)	yes	U	no	no	no	no	yes	yes	yes	yes	yes	yes	7
Cruser (2012)	yes	yes	no	no	no	yes	yes	yes	yes	yes	yes	yes	9
Vismara (2012)	yes	yes	no	no	no	yes	no	yes	yes	yes	yes	yes	8
Licciardone (2013a)	yes	yes	U	no	U	yes	no	no	yes	yes	U	yes	6
Licciardone (2013b)	yes	yes	U	no	U	yes	yes	no	yes	yes	yes	yes	8
Belz (2015)	yes	yes	no	no	no	yes	yes	yes	yes	yes	U	yes	8
Hensel (2015)	yes	yes	no	no	no	no	yes	yes	no	yes	no	yes	6
Schwerla (2015)	yes	yes	U	no	no	yes	yes	yes	no	yes	U	yes	7
Licciardone (2016)	yes	yes	U	no	U	yes	yes	yes	yes	yes	yes	yes	9

*this criterion was scored ‘no’ if a significant between-group difference for at least one variable was present

**single-session intervention

ITT, intention-to-treat; NA, not applicable; U, unsure

## Discussion

The current literature review was to some extent different from previous reviews assessing the effectiveness of osteopathic care for spinal complaints. Previous reviews were aimed at a specific spinal area such as non-specific LBP [15, 17, 18] or chronic non-specific neck pain [16]. Conversely, for the current review, randomized controlled trials examining the effect of osteopathic care for spinal complaints independently from their location and from the pain duration were considered. Giving consideration to an ‘authentic’ osteopathic intervention and thus considering the ‘real-world’ osteopathic practice, only studies in which the treating osteopathic practitioner had a free choice of determining the treatment based on the clinical judgement were eligible for inclusion in the reviews by Franke et al. [15, 16]. For the current review, we defined studies applying such an approach as being ‘custom tailored’. Though recognizing this pragmatic approach representing ‘real-world’ osteopathic practice, we also included studies applying a ‘standardized’ or ‘semi-standardized’ protocol in order to assess possible differences in effectiveness between the different treatment protocols. For the outcome ‘pain’, significant between-group differences were observed in eight of nine studies applying a ‘custom tailored’ approach, while these figures were seven of seven studies for those applying a ‘semi-standardized’ protocol. For functional status, significant between-group differences were identified in five of seven studies and three of seven studies applying a ‘custom tailored’ and ‘semi-standardized’ protocol, respectively. In the one study with a ‘standardized’ osteopathic intervention, a non-significant between group difference was observed.

In general, the results showed mixed findings related to the impact of osteopathic care for spinal complaints on the primary outcomes ‘pain’ and ‘functional status’. In some studies, osteopathic care was found to be effective compared to control groups. In other studies, significant improvements in pain and functional status were observed in the osteopathic care groups, but no significant between-group differences were identified. Finally, in some studies neither significant within-group or between-group effects were found. For pain, significant between-group differences in favor of osteopathic care were observed in all European studies, while for functional status this was the case in all but one study. The findings from the US studies of the impact of osteopathic care on pain and functional status were less homogeneous. For ‘functional status’, differences in treatment protocols may probably explain the variation in effects of osteopathic care between European and US studies. Five of eight European studies evaluating functional status applied a ‘custom tailored’ protocol, all showing osteopathic care to be effective. Contrary, in the two US studies with such a protocol, osteopathic care resulted in a non-significant improvement in functional status. For the outcome measure ‘pain’, the association with treatment protocol was less clear. Thus, it appears that other elements than the treatment protocol are likely to explain the differences in impact of osteopathic care for spinal complaints between European and US studies. A possible explanation is the, largely for historical reasons, different evolution of osteopathy in Europe vs. the US, creating two professionals streams with two different scopes of practice. Osteopathy in Europe thus developed within a more limited scope of practice, concentrating more on manual techniques [13]. Mixed findings were also found for the secondary outcomes of interest ‘medication use’ and ‘health status’.

The results related to the outcomes ‘pain’ and ‘functional status’ were evaluated taking into account four levels of effectiveness. We are aware that presenting the findings in such a way is rather unusual. This approach enabled us to compare the findings based on different variables such as study protocol (custom tailored, semi-standardized, and standardized) and geography (European vs. US studies). In addition, this method appeared to be appropriate as the current study consisted of a qualitative evidence synthesis. This type of literature is now acknowledged as a necessary and valuable type of information to answer health service research questions [47]. The studies were characterized by a number of clinical and methodological differences including different treatment protocols, differences in treatment strategies in the comparison groups (e.g. no intervention, sham manipulation, exercise, physiotherapy), differences in treatment duration, absence of follow-up in some studies, different target populations (e.g. pregnant women, obese females, women post-partum, diabetes patients), and differences in sample sizes. Such differences hinder the comparison of the findings across studies. The extent of methodological heterogeneity between the included studies was not quantified since this is useful for the interpretation of a meta-analysis [48] and it was not the aim of the current study to perform such analysis. Studies based on more homogeneous methodologies would enable to better compare the findings of trials. Nevertheless, we decided to include studies irrespective of their differences in methodologies to provide a broad overview of the impact of osteopathic care in the treatment of spinal complaints. Several of these methodological shortcomings were already addressed in previous literature reviews [15–18] examining the impact of osteopathic care for spinal complaints, but also in reviews of the effect of osteopathic care for other conditions such as irritable bowel syndrome [49], lower urinary tract symptoms in women [50], pediatric conditions [20], and length of stay in preterm infants [51].

A number of limitations need to be addressed. First, only studies published from 1995 on were eligible for inclusion. It is thus possible that we have missed relevant papers prior to 1995. Second, there is a risk of reporting bias, since only studies published in English, French, German, or Dutch were considered. Third, the review focused on the outcomes ‘pain’, ‘functional status’, ‘medication use’, and ‘health status’. We are aware that other outcomes may also be worthwhile to study such as the impact of osteopathic care on productivity and satisfaction with treatment. Fourth, five studies were derived from the ‘grey literature’ which is not peer-reviewed. However, since only randomized controlled study designs were considered, these studies met this inclusion criterion. Moreover, the quality of these papers was also assessed using the checklist as recommended by the Cochrane Back Review Group. Fifth, assessment of the risk of bias was performed by one researcher, while the ‘Cochrane Back and Neck Group’ indicate that risk of bias in the studies should be independently assessed by at least two authors [26].

In conclusion, the findings of the current literature review suggested that osteopathic care may improve pain and functional status in patients suffering from spinal complaints. A clear distinction was observed between studies conducted in the US and those in Europe, in favor of the latter. Today, no clear conclusions of the impact of osteopathic care for spinal complaints can be drawn. Further studies with larger study samples also assessing the long-term impact of osteopathic care for spinal complaints are required to further strengthen the body of evidence.

## Appendix 1 Search strategy Medline (Pubmed)

1. Low back pain [MeSH]

2. Back pain [MeSH]

3. Neck pain [MeSH]

4. Pelvic pain [MeSH]

5. Pelvic girdle pain [MeSH]

6. Spine [MeSH]

7. Spinal [title/abstract]

8. #1 OR #2 OR #3 OR #4 OR #5 OR #6 OR #7

9. Manipulation, osteopathic [MeSH]

10. Manipulation, spinal [MeSH]

11. Osteopathic medicine [MeSH]

12. Osteopathy [title/abstract]

13. Osteopathic [title/abstract]

14. Manipulative [title/abstract]

15. Manipulation [title/abstract]

16. #9 OR #10 OR #11 OR #12 OR #13 OR #14 OR #15

17. Comparative effectiveness research [MeSH]

18. Pragmatic clinical trial [MeSH]

19. Effectiveness [title/abstract]

20. Efficacy [title/abstract]

21. #17 OR #18 OR #19 OR #20

22. #8 AND #16 AND #21

Limits: publication date 01/01/1995–31/05/2018; humans

## Supporting information

S1 Table PRISMA 2009 checklist. (DOC)
