# Correction: Osteopathic care for spinal complaints: A systematic literature review

**DOI:** 10.1371/journal.pone.0221140

**Published:** 2019-08-08

**Authors:** Nick Verhaeghe, Janne Schepers, Patrick van Dun, Lieven Annemans

In the Primary outcome-functional status subsection of the Results section, there is an error in the sixth sentence. The correct sentence is: In the study by Hensel et al. [29], osteopathic care was found to significantly improve functional status.

In the Primary outcome-functional status subsection of the Results section, there is an error in the last sentence. The correct sentence is: In another US study [30], functional status also significantly improved in the osteopathic care group compared to controls (Table 5).

There is an error in Table 5. The treatment effect of the Licciardone (2010) study should be noted as having a significant between-group difference.

**Table 5:** Effectiveness of osteopathic care related to the outcome ‘functional status’.

Study	Treatment protocol	treatment effect			
		OT worse than controls	non-significant improvement OT	signif. improvement OT/no signif. between-group difference	significant between-group difference
**EUROPEAN STUDIES**					
Peters (2006)	custom				x
Heinze (2006)	custom				x
Recknagel (2008)	custom				x
Schwerla (2015)	custom				x
Belz (2015)	custom				x
UK BEAM (2004)	semi-standardized				x
Chown (2008)	semi-standardized			x	
Vismara (2012)	semi-standardized				x
**US STUDIES**					
Andersson (1999)	custom		x		
Licciardone (2003)	custom		x		
Cruser (2012)	semi-standardized			x	
Licciardone (2013b)	semi-standardized		x		
Hensel (2015)	semi-standardized			OT vs. PUT	OT vs. UOC
Licciardone (2016)	semi-standardized		x		
Licciardone (2010)	standardized				x

OT, osteopathic treatment; PUT, placebo ultrasound treatment; UOC, usual obstetrical care
